# Trends and Patterns of Perfluoroalkyl Substances in Blood Plasma Samples of Bald Eagle Nestlings in Wisconsin and Minnesota, USA

**DOI:** 10.1002/etc.4864

**Published:** 2020-10-20

**Authors:** Cheryl R. Dykstra, William T. Route, Kelly A. Williams

**Affiliations:** ^1^ Raptor Environmental, West Chester Ohio USA; ^2^ US National Park Service, Great Lakes Inventory and Monitoring Network Ashland Wisconsin USA; ^3^ Department of Biological Sciences, Ohio University, Athens Ohio USA

**Keywords:** Bald eagle, Great Lakes, *Haliaeetus leucocephalus*, Perfluoroalkyl substances, Contaminants

## Abstract

We analyzed concentrations and trends of perfluoroalkyl substances (PFAS) in blood plasma samples of bald eagle (*Haliaeetus* *leucocephalus*) nestlings at 6 study areas in the upper Midwest of the United States, 2006 to 2015, and long‐term trends at 2 Lake Superior (USA/Canada) sites, 1995 to 2015. Nestling blood plasma concentrations of the sum of 15 PFAS analytes (∑PFAS) differed among study areas and were highest at the 3 industrialized river sites: pools 3 and 4 of the Mississippi River (pools 3 + 4; geometric mean [GM] = 754 μg/L; range = 633–2930), the Mississippi National River and Recreation Area (GM = 687 μg/L; range = 24–7371), and the lower St. Croix National Scenic Riverway (GM = 546 μg/L; range = 20–2400). Temporal trends in ∑PFAS in nestling plasma differed among study areas; concentrations decreased at pools 3 + 4, Mississippi National River and Recreation Area, and lower St. Croix National Scenic Riverway, but not at the most remote sites, the upper St. Croix River and Lake Superior. Overall, perfluorooctanesulfonate **(**PFOS) was the most abundant analyte at all study areas, and perfluorodecanesulfonate (PFDS) the second most abundant at industrialized river sites although not at Lake Superior; concentrations of both these analytes declined from 2006 to 2015 over the study area. In addition, nestling age significantly influenced plasma concentrations of ∑PFAS and 7 of the 12 analytes. For these analytes, concentrations increased by 1 to 2%/d as nestlings grew, indicating that age should be considered when using nestling plasma to assess PFAS. *Environ Toxicol Chem* 2021;40:754–766. © 2020 The Authors. *Environmental Toxicology and Chemistry* published by Wiley Periodicals LLC on behalf of SETAC.

## Graphical abstract

Concentrations of perfluoroalkyl substances (PFAS) in blood plasma of bald eagle nestlings declined at 3 industrialized river sites, but not at a remote river site or Lake Superior. Nestling age significantly influenced plasma concentrations of total PFAS and most analytes.

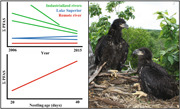

## INTRODUCTION

Perfluoroalkyl and polyfluoroalkyl substances (PFAS) are found in nonstick cookware, water‐repellent and stain‐repellent formulations, fire‐fighting foams, pesticides, surfactants, and other applications (Custer et al. 2013; Route et al. 2014a). Since their introduction in the 1950s, they have been manufactured in great quantities and are widely distributed, even to areas where they were never used. Water, sediment, and biota from diverse locations worldwide are contaminated by these persistent, bioaccumulative compounds (Stahl et al. 2011; Remucal 2019).

Perfluoroalkyl and polyfluoroalkyl substances have been linked to negative impacts in various species, including humans. In laboratory studies, PFAS affect the function of the endocrine system in birds and humans (Weiss et al. 2009; Tartu et al. 2014), the liver, lungs, kidneys, and immune system, and the health and behavior of offspring of exposed females (Stahl et al. 2011). Field studies have reported effects on metabolic rate (Blévin et al. 2017), reproductive rate of birds (Custer et al. 2012, 2014; Tartu et al. 2014), and human behavior (Gump et al. 2011), although the interpretation of these results is sometimes confounded by the presence of other toxic bioaccumulative contaminants (Custer et al. 2019).

Perfluorooctanesulfonate (PFOS), which is among the most commonly studied PFAS due to its abundance and ubiquity in biota, accounted for the majority of PFAS in various field‐collected organisms (Kannan et al. 2005; Stahl et al. 2014; Remucal 2019), including fish in Midwestern rivers in the United States (Ye et al. 2008), and eggs of peregrine falcons (*Falco peregrinus*; Vorkamp et al. 2019), great tits (*Parus major*; Groffen et al. 2019), and Great Lakes Caspian terns (*Hydroprogne caspia*; Su et al. 2017) and bald eagles (*Haliaeetus leucocephalus*; Wu et al. 2020). Chronic exposure to PFOS under experimental conditions is associated with failure to hatch, decreased weight gain (Dennis et al. 2020), decreased survival, and lower body mass (Bursian et al. 2020).

Because of the widespread use and toxicity of PFAS, it is important to monitor and understand their distribution and effects in biota, to inform regulatory decisions and wildlife management and conservation efforts. Bald eagles serve as excellent biosentinels for monitoring a variety of biomagnifying toxic compounds, due to their position at the top of the aquatic food web (Elliott and Norstrom 1998; Dykstra et al. 2001; Elliott and Harris 2001–2002; Buck et al. 2005; Cesh et al. 2010; Wierda et al. 2016; Wu et al. 2020). Monitoring contaminant concentrations in nestlings is particularly valuable for detecting local levels and sources of compounds, because the adult eagles tend to forage in a relatively small area surrounding their nest (Stalmaster 1987; Garrett et al. 1993), and concentrations in the eaglets thus reflect local conditions (Elliott et al. 2009; Route et al. 2019). Bald eagle nestlings have already served as bioindicators for legacy contaminants such dichlorodiphenyldichloroethylene (DDE), polychlorinated biphenyls (PCBs), and mercury (Dykstra et al. 2001, 2019; Elliott et al. 2009; Cesh et al. 2010; Wierda et al. 2016) as well as emerging contaminants such as polybrominated diphenyl ethers (PBDEs; Venier et al. 2010; Route et al. 2014b; Guo et al. 2018) and PFAS (Kannan et al. 2001; Route et al. 2014a), particularly in the Laurentian Great Lakes (USA/Canada) ecosystem.

Great Lakes biota consistently have higher loads of persistent organic pollutants than the same species in much smaller lakes (often termed “inland lakes”) in the region (Giesy et al. 1995; Stahl et al. 2013, 2014). An early assessment of PFOS concentrations in the Great Lakes region reported that bald eagle nestling plasma concentrations of this analyte averaged 330 ng/mL in the early 1990s, although most samples were from inland lakes or rivers rather than from the Great Lakes shores (Kannan et al. 2001). Biomagnification accounted for enhanced concentrations of PFOS that were 10 to 20 times higher in Great Lakes eagles than in their prey species (Kannan et al. 2005). More recently, bald eagle nestlings along the south shore of Lake Superior (USA/Canada) had moderate levels of total PFAS, averaging 490 to 550 ng/mL in nestling plasma (Route et al. 2014a), and bald eagle eggs in the Great Lakes region had median concentrations of 174 ng/g, with higher levels on the Great Lakes shores than in inland areas (Wu et al. 2020).

In addition to the Great Lakes, urban areas and other areas affected by anthropogenic activity often have higher levels of PFAS in sediment, water, and biota (Stahl et al. 2014; Remucal 2019). A large‐scale multimedia study in Canada detected elevated concentrations of PFOS associated with urbanization, especially in urban areas along the Great Lakes; river sites with relatively high concentrations were influenced by urban development or wastewater treatment plants (Gewurtz et al. 2013). Similarly, double‐crested cormorants (*Phalacrocorax auritus*), dolphins (*Tursiops truncatus*), and northern pike (*Esox lucius*) associated with industrialized development or wastewater treatment plants exhibited higher levels of PFAS compared with those sampled in more remote regions (De Silva et al. 2016).

Although bald eagles were removed from the Endangered Species list in 2007 (US Fish and Wildlife Service 2007), they are still protected under the Bald and Golden Eagle Protection Act (16 U.S.C. 668‐668c), and continued monitoring of population trends and contaminant loads is recommended to safeguard their recovery and to serve as vital indicators of aquatic ecosystem health. As a follow‐up to our earlier study (Route et al. 2014a), we report concentrations and temporal trends for PFAS in bald eagles in 6 study areas with different hydrological regimes, prey bases, and anthropogenic impacts. We predicted that overall PFAS concentrations would be highest at urban sites or sites influenced by anthropogenic development, and that concentrations would decrease over the course of the study at most sites.

## MATERIALS AND METHODS

### Collection of samples

We collected blood samples from bald eagle nestlings at 6 study areas in northern Wisconsin and adjacent areas of Minnesota: the Apostle Islands National Lakeshore, the southern shore of Lake Superior in Wisconsin, the upper St. Croix National Scenic Riverway, the lower St. Croix National Scenic Riverway, the Mississippi National River and Recreation Area, and pools 3 and 4 of the Mississippi River (Pools 3 + 4; Route et al. 2014a; Supplemental Data, Figure S1). Nests classified as Lake Superior southern shore were located within 8 km of the southern edge of Lake Superior; for all other study areas, nests were included if they were within 0.5 km of the protected area boundary. Because eagle pairs may use different nest structures in different years, we defined a nesting territory as the area that contained all the nests within the home range of a mated pair (as in Steenhof and Newton 2007); generally, nests within one territory were within 1 km of each other.

From May to June, 2006 to 2015, a qualified tree‐climber accessed nests when young were 5 to 9 wk old and lowered them to the ground. Nestlings were banded, weighed, and measured (8th primary, footpad, hallux, bill depth, and culmen). Nestlings were aged by the length of the 8th primary (Bortolotti 1984) and sexed using genetic analysis. We collected ≤10 mL of blood from the brachial vein of all nestlings in each nest (1–4), unless they were too young or too old to safely sample. We transferred the samples to 10‐mL Vacutainers, and stored them on blue‐ice until the end of the day (a method not currently recommended for PFAS but commonly used during our study). We then centrifuged the samples at 1200 rpm to separate plasma from red blood cells. For each nest, a single sample was chosen for PFAS measurements (arbitrarily in 2006, randomly thereafter). We used a sterile glass pipette to transfer 1.0 mL of plasma to a polypropylene vial as the sample aliquot. Glass pipettes were previously baked at 650 °F (343 °C) to remove chemical residues. A sample of stock syringes, needles, Vacutainers, and vials that we used were tested by the 3M Environmental Laboratory (Maplewood, MN, USA) and verified to be free of PFAS chemicals. Plasma samples were immediately frozen, and kept frozen until delivery to the Wisconsin State Laboratory of Hygiene (Madison, WI, USA) for analysis.

For analysis of long‐term trends of PFAS in nestlings along the Lake Superior shore (Apostle Islands National Lakeshore and Lake Superior southern shore study areas), we also analyzed archived plasma samples (*n* = 10 for Lake Superior southern shore, 1995–1998; *n* = 4 for Apostle Islands National Lakeshore, 1998–2002; Supplemental Data, Table S1). These archived samples were collected and stored using the methods just described by the Wisconsin Department of Natural Resources.

Two hundred sixty‐one of the samples collected from 2006 to 2011 were previously analyzed using a different statistical method (mixed effects models in a Bayesian framework; Route et al. 2014a). These samples are included in the present study for updated, longer term analyses that allow more thorough assessment of concentrations and trends due to larger sample sizes.

### Laboratory procedures

Analytical techniques have already been described in detail previously (Route et al. 2014a). Briefly, Wisconsin State Laboratory of Hygiene performed all measurements using high‐performance liquid chromatography tandem mass spectrometry with quantification using turbo ion spray triple quadruple mass spectrometer in the negative ionization mode. Quality control consisted of reagent blanks, method blanks, and spiked samples of known quantity for calibrations for every batch of 10 samples. With a subset of our samples we conducted a blind, interlaboratory comparison with the US Environmental Protection Agency laboratory in Research Triangle Park (NC, USA) and the 3M Environmental Laboratory, and concluded that PFAS analytes found in high concentrations could be consistently measured with high reproducibility between laboratories. The less abundant analytes varied more widely in magnitude between laboratories, but they trended in the same direction (Route et al. 2014a).

### Statistical analyses

*Sum of PFAS*. Each sample contained concentrations of up to 15 PFAS compounds (2–15 analytes; ∑PFAS; compounds and abbreviations shown in the Supplemental Data, Table S2). Because many of these concentrations were below the limit of quantitation (LOQ; 0–98% of the samples for each analyte), we calculated the sum of the PFAS analytes using the Kaplan–Meier techniques (Helsel 2012). The Kaplan–Meier sums for each sample were calculated in R Ver 3.4.3 (R Core Team 2017) with the function *cenfit* in the package NADA Ver 1.6‐1.1 (Lee 2017) by multiplying the Kaplan–Meier mean by the number of analytes (Helsel 2012).

We used a mixed effects model in the package *nlme* Ver 3.1‐141 (Pinheiro et al. 2019) to compare Kaplan–Meier sums for samples collected between 2006 and 2015 among the 6 study areas. We examined long‐term trends at Lake Superior southern shore (1995–1998, and 2007–2015) and Apostle Islands National Lakeshore (1998, 2000, 2002, 2006–2015) in separate analyses. We used nesting territory as a random effect rather than individual nest because samples within a nesting territory are not considered independent. In addition, the use of territory as a random effect helped to control for spatial autocorrelation. Nestling age at sampling was included as a covariate because previous studies have indicated that PFAS concentrations can increase as nestlings age (Bustnes et al. 2013; Route et al. 2014a; Løseth et al. 2019). We included study area, year, the interaction between study area and year, and nestling age at sampling as fixed effects and territory as a random effect. We plotted the residuals against *XY* coordinates to check for spatial autocorrelation. Models were fit using generalized least squares and mixed effects models with maximum likelihood, and then compared using the bias‐corrected Akaike information criteria (AIC_*c*_; Burnham and Anderson 2002) with the function *model.sel* in package MuMIN (Barton 2016) to determine the fixed and random structure of each model. The AIC_*c*_‐selected model was refit using restricted maximum likelihood (REML), and inferences were made from this model (Zuur et al. 2009). If we detected significant differences in our REML model among study areas, we then used Tukey's post hoc comparison tests in the package *lsmeans* (Lenth 2016) to determine differences, considering *p* < 0.05 to indicate significant differences among study areas. We calculated geometric means by back‐transforming the least square means using 10^β^ Table 1.

**Table 1:** Mean and range (in μg/L) of sum perfluoroalkyl substances (ΣPFAS) and PFAS analytes in blood plasma of bald eagle nestlings at 6 study areas in Wisconsin and Minnesota

	APIS		LSSS		USACN		LSACN		MISS		Pools 3 + 4	
Analyte^a^	Mean	Range	Mean	Range	Mean	Range	Mean	Range	Mean	Range	Mean	Range
∑PFAS^a^, ^b^	241.3	96–1420	175.6	79–382	29.9	9–205	545.5	20–2400	686.5	24–7371	753.7	633–2930
PFOS^a^	135.2	69–830	122.5	47–290	19.5	7.5–180	384.5	10–2400	540.9	13–4200	571.0	440–1400
PFDS	2.8	LOQ–100	3.2	0.7–32	0.9	LOQ–20	144.8	6.2–860	119.8	0.4–4100	368.2	130–1400
PFDA	21.6	LOQ–77	10.0	4.4–29	2.5	1.1–7.1	12.5	2.4–30	17.2	2.2–85	15.9	LOQ–37
PFUnA^a^	40.2	17–110	21.0	7.1–55	2.9	1.1–6.4	6.5	2–19	7.7	1.7–33	7.2	2.3–65
PFDoA^a^	9.0	4–27	5.5	2–14	0.6	0.21–1.2	4.2	0.9–18	6.3	0.5–33	5.1	2.4–31
PFNA^a^	53.7	24–160	13.4	2.6–83	2.5	1.1–8.3	3.0	1–12	4.1	0.8–19	3.3	1.2–11
PFTrA^a^	18.4	8–63	8.7	3.6–48	0.9	0.13–5.8	2.4	0.6–14	2.3	0.5–14	1.9	0.9–12
PFHpS	1.1	0.6–5.4	0.8	0.2–1.8	0.2	LOQ–2.9	1.5	LOQ–4.4	3.4	0.2–16	4.8	2–11
PFHxS	1.0	0.3–8.6	0.9	LOQ–2.7	0.3	LOQ–9.1	1.2	LOQ–8.3	4.0	0.3–47	4.7	0.8–26
PFTeA	2.5	0.8–19	1.3	0.4–16	0.4	LOQ–2.4	1.5	0.2–14	1.7	0.3–310	1.0	LOQ–14
PFOA	2.2	0.2–14	1.0	LOQ–5.3	0.2	LOQ–0.8	0.2	LOQ–10	0.5	LOQ–10	0.5	0.1–1.2
PFBA	0.03	LOQ–22	0.08	LOQ–0.8	0.06	LOQ–0.9	0.3	LOQ–46	0.5	LOQ–78	0.3	LOQ–5.6

^a^ Means for analytes are geometric means calculated from the mixed effects models. Means for unmarked analytes are estimated means from the frailty models for censored data with lognormal distribution.

^b^ ∑PFAS is the sum of up to 15 analytes of PFAS calculated using the Kaplan–Meier technique described in Helsel (2012).

Study site abbreviations: APIS = Apostle Islands National Lakeshore; LSSS = southern shore of Lake Superior in Wisconsin; USACN = upper St. Croix National Scenic Riverway; LSACN = lower St. Croix National Scenic Riverway; MISS = Mississippi National River and Recreation Area; Pools 3 + 4 = pools 3 and 4 of the Mississippi River. PFOS = perfluorooctanesulfonate; PFDS = perfluorodecanesulfonate; PFDA = perfluorodecanoate; PFUnA = perfluoroundecanoate; PFDoA = perfluorododecanoate; PFNA = perfluorononanoate; PFTrA = perfluorotridecanoate; PFHpS = perfluoroheptanesulfonate; PFHxS = perfluorohexanesulfonate; PFTeA = perfluorotetradecanoate; PFOA = perfluorooctanoate; PFBA = perfluorobutanoate; LOQ = lower limit of quantification.

#### Individual analytes

For PFAS with no samples <LOQ (PFOS, perfluoroundecanoate [PFUnA], perfluorododecanoate [PFDoA], perfluorononanoate [PFNA], and perfluorotridecanoate [PFTrA]), we used mixed effects models as just described in the *Sum of PFAS* section to compare samples collected between 2006 and 2015 at 6 study areas (Apostle Islands National Lakeshore, lower St. Croix National Scenic Riverway, Lake Superior southern shore, Mississippi National River and Recreation Area, pools 3 + 4, and upper St. Croix National Scenic Riverway). We used a log_10_ transformation of analyte concentration to meet the assumptions of normality.

For analytes with <80% of samples below the LOQ (perfluorodecanesulfonate [PFDS], perfluorodecanoate [PFDA], perfluoroheptanesulfonate [PFHpS], perfluorohexanesulfonate [PFHxS], perfluorotetradecanoate [PFTeA], perfluorooctanoic acid [PFOA] and perfluorobutanoate [PFBA]), we followed the recommendations of Helsel (2012). We fit a parametric survival mixed effect model (frailty model; Govindarajulu et al. 2011) using function *survreg* in the package *survival* (Ver 2.38; Therneau 2015) and used the *frailty* function to add territory as a random effect with a gamma distribution and an expectation maximization algorithm. The expectation maximization method uses unobserved latent variables to create the expectation of the log‐likelihood and is used when equations cannot be solved directly. The combination of the gamma distribution and expectation maximization method was selected as the best for defining the random effect term in our models. A left‐censored survival object for each analyte concentration and the respective LOQ was created for each model. The mixed effect model was fit with a lognormal distribution. We checked the standard residual plots to ensure model assumptions were met. We estimated means from predicted values on the response scale from the AIC_*c*_‐selected model (Table 1).

We did not conduct individual analyses for 3 analytes with high numbers of samples (90–98%) below the LOQ: perfluoropentanoate (PFPA), perfluorohexanoate (PFHxA), and perfluoroheptanoate (PFHpA; Supplemental Data, Table S3). We conducted all analyses in R Ver 3.4.3 (R Core Team 2017).

## RESULTS

### Samples collected

From 2006 to 2015, we collected 375 nestling samples from 162 nesting territories in the 6 study areas. The number of samples for each study area varied due to the size of the eagle population, nest occupancy rates, and funding sources. More samples were collected at Mississippi National River and Recreation Area (*n* = 141) than at other study areas (lower St. Croix National Scenic Riverway, *n* = 64; upper St. Croix National Scenic Riverway, *n* = 64; Apostle Islands National Lakeshore, *n* = 57; pools 3 + 4, *n* = 33; Lake Superior southern shore, *n* = 16; Supplemental Data, Table S1). Nestling age ranged from 19 to 71 d.

### Sum of PFAS

The ∑PFAS in nestling plasma was highest at pools 3 + 4, lowest at upper St. Croix National Scenic Riverway, and differed among study areas (Table 1). The AIC_*c*_‐selected model of ∑PFAS in plasma included the interaction between study area and year and nestling age as a covariate (Supplemental Data, Figure S2). The model suggested significant differences among study areas. Tukey's post hoc comparisons indicated that ∑PFAS in nestling plasma was greater at lower St. Croix National Scenic Riverway, Mississippi National River and Recreation Area, and pools 3 + 4 than at Apostle Islands National Lakeshore, Lake Superior southern shore, and upper St. Croix National Scenic Riverway (all *p* < 0.001; Supplemental Data, Figure S3). The ∑PFAS in nestling plasma was greater at Apostle Islands National Lakeshore and Lake Superior southern shore compared with upper St. Croix National Scenic Riverway (both *p* < 0.001). In addition, ∑PFAS concentration increased by 1.2 μg/L/d (0.0068 ± 0.0016 [standard error (SE)] on a log_10_ scale) with nestling age (*t* = 4.35, *p* < 0.001).

**Figure 1:**
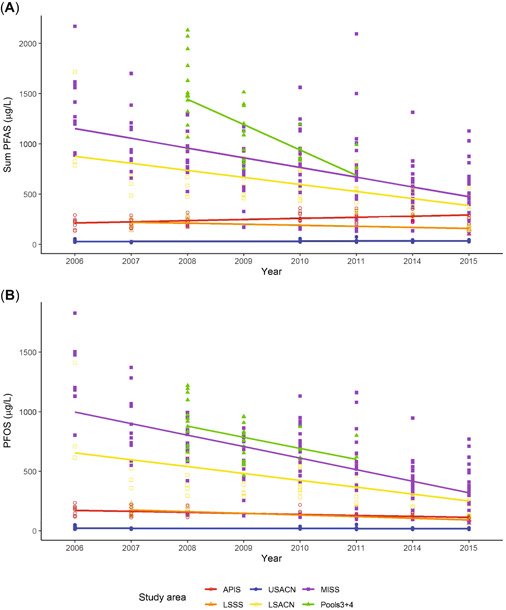
Temporal trends in perfluoroalkyl substances (PFAS) concentrations: (**A**) ∑PFAS in μg/L and (**B**) perfluorooctanesulfonate (PFOS) concentrations (μg/L) in plasma of nestling bald eagles sampled at 6 study areas in northern Wisconsin and adjacent areas of Minnesota, 2006 to 2015. Concentrations are predicted values from the models with study area × year interaction, nestling age as a covariate, and territory as a random factor. For (**A**), trends at Mississippi National River and Recreation Area, lower St. Croix National Scenic Riverway, and pools 3 + 4 are significant; others are not but lines are shown for illustrative purposes. For (**B**), lines for individual study area are shown for illustrative purposes. Overall PFOS declined over the study period (*p* = 0.057). APIS = Apostle Islands; LSSS = the south shore of Lake Superior in Wisconsin; USACN = upper Saint Croix National Scenic River; LSACN = lower Saint Croix National Scenic River; MISS = Mississippi National River and Recreation Area; and Pools 3 + 4 = pools 3 and 4 of the Mississippi River.

Trends in ∑PFAS in nestling plasma differed among study sites. Although we found no overall trend over time (β = –0.0177 ± 0.01; *t* = 1.68, *p* = 0.09), 3 interactions between study area and year were significant, indicating trends at these study areas. The ∑PFAS in nestling plasma decreased at lower St. Croix National Scenic Riverway (10.1%; β = –0.0419 ± 0.01 [SE]; *t* = –3.74, *p* < 0.001), Mississippi National River and Recreation Area (10.8%; β = –0.0447 ± 0.007; *t* = –6.25, *p* < 0.001) and pools 3 + 4 (34.4%, β = –0.1286 ± 0.051; *t* = 2.5, *p* = 0.01; Figure 1A). However, the trend at pools 3 + 4 should be considered tentative because samples were few and unevenly distributed: *n* = 15 in 2008, 12 in 2009, 4 in 2010, and 2 in 2011.

#### Long‐term trends at Lake Superior

There were no significant long‐term trends in ∑PFAS in nestling plasma by year at Lake Superior southern shore (*t* = –0.18, *p* = 0.86) or at Apostle Islands National Lakeshore (*t* = 1.19, *p* = 0.24). Nestling age was included in the AIC_*c*_‐selected model of ∑PFAS at Apostle Islands National Lakeshore and indicated that ∑PFAS increased with nestling age by 1.4%/d (0.0058 ± 0.003 on the log_10_ scale; *t* = 2.0, *p* = 0.05).

### Individual analytes

#### General patterns

The analytes with the greatest concentrations in nestling plasma were PFOS and PFDS (Table 1). Concentrations in nestling plasma of both PFOS and PFDS were lowest at upper St. Croix National Scenic Riverway, and highest at Pools 3 + 4, followed by Mississippi National River and Recreation Area and lower St. Croix National Scenic Riverway. The nestlings at Lake Superior sites (Apostle Islands National Lakeshore and Lake Superior southern shore) had lower PFOS concentrations and much lower PFDS concentrations. Nonetheless, nestlings at Apostle Islands National Lakeshore had the greatest concentrations for 7 of the 12 analytes reported, including PFOA, PFDA, PFUnA, PFNA, and PFDoA (Table 1). Eagles at upper St. Croix National Scenic Riverway had the lowest mean concentrations for 11 of the 12 analytes.

#### PFOS

The most abundant analyte in nestling bald eagle plasma samples was PFOS, with geometric means ranging from 19.5 μg/L at upper St. Croix National Scenic Riverway to 571 μg/L at Pools 3 + 4. The AIC_*c*_‐selected model included the interaction between study area and year, and nestling age as a covariate. PFOS concentration in nestling plasma increased by 1.9%/d with nestling age (0.00799 ± 0.0016 [SE] on the log_10_ scale; *t* = 5.10, *p* < 0.001) and decreased by 4.8%/yr (0.0202 ± 0.01 log_10_ scale; *t* = −1.91, *p* = 0.057; Figure 1B). Tukey post hoc comparisons indicated significant differences among study areas (Supplemental Data, Figure S4). Concentrations of PFOS in nestling plasma at Pools 3 + 4, Mississippi National River and Recreation Area, and lower St. Croix National Scenic Riverway were greater than those at Apostle Islands National Lakeshore, Lake Superior southern shore, and upper St. Croix National Scenic Riverway (all *p* < 0.001), and concentrations in nestling plasma at Apostle Islands National Lakeshore and Lake Superior southern shore were also greater than those at upper St. Croix National Scenic Riverway (both *p* < 0.001; *df* = 161 for Apostle Islands National Lakeshore, *df* = 156 for all others).

#### PFDS

**Figure 2:**
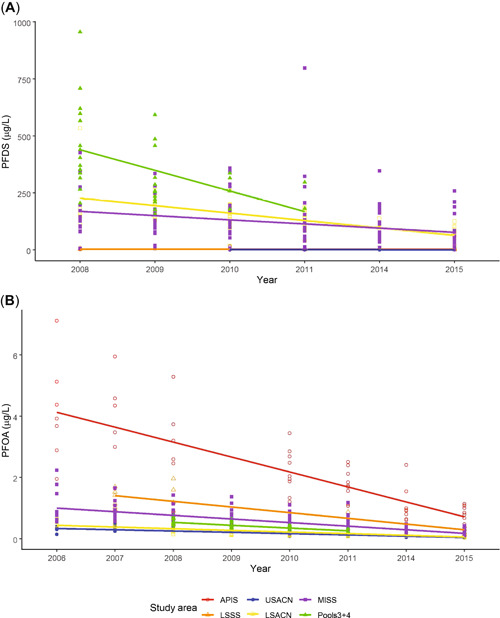
Temporal trends of (**A**) perfluorodecanesulfonate (PFDS; μg/L) and (**B**) perfluorooctanoic acid (PFOA; μg/L) in plasma of nestling bald eagles sampled at 6 study areas in northern Wisconsin and adjacent areas of Minnesota, 2006 to 2015. For (**A**), concentrations are predicted values from the frailty model with study area, year, and nestling age as predictors and territory as a random effect. Lines for individual study area are shown for illustrative purposes. Overall PFDS declined significantly over the study period (*p* < 0.001). For (**B**), concentrations are predicted values from the frailty model with study area and year as predictors and territory as a random effect. Lines for individual study area are shown for illustrative purposes. Overall PFOA declined significantly over the study period (*p* < 0.001). APIS = Apostle Islands; LSSS = the south shore of Lake Superior in Wisconsin; USACN = upper Saint Croix National Scenic River; LSACN = lower Saint Croix National Scenic River; MISS = Mississippi National River and Recreation Area; and Pools 3 + 4 = pools 3 and 4 of the Mississippi River.

The second most abundant analyte in nestling eagle plasma was PFDS, with means ranging from 0.9 μg/L at upper St. Croix National Scenic Riverway to 368.2 μg/L at Pools 3 + 4. Because 4 samples were below the LOQ (Apostle Islands National Lakeshore *n* = 1; upper St. Croix National Scenic Riverway *n* = 3), we used the frailty model. The PFDS concentrations in nestling plasma declined by year (β = −0.1630 ± 0.0169 [SE], χ^2^ = 93.08, *p* < 0.001; Figure 2A) and increased with nesting age (β = 0.0144, χ^2^ = 8.36, *p* = 0.004). The PFDS concentrations in nestling plasma were highest at Pools 3 + 4, followed by lower St. Croix National Scenic Riverway and Mississippi National River and Recreation Area (Table 1). Post hoc comparisons indicated significant differences among study areas. Concentrations of PFDS in nestling plasma at Pools 3 + 4, Mississippi National River and Recreation Area, and lower St. Croix National Scenic Riverway were greater than those at Apostle Islands National Lakeshore, Lake Superior southern shore, and upper St. Croix National Scenic Riverway (all *p* < 0.05), and concentrations at Apostle Islands National Lakeshore and Lake Superior southern shore were also greater than those at upper St. Croix National Scenic Riverway (both *p* < 0.05).

#### PFOA

The PFOA concentrations in nestling plasma were generally low, with many samples below the LOQ (lower St. Croix National Scenic Riverway *n* = 24, Lake Superior southern shore *n* = 2, Mississippi National River and Recreation Area *n* = 11, upper St. Croix National Scenic Riverway *n* = 34). The PFOA concentrations in nestling plasma declined by year (β = –0.1741 ± 0.014 [SE], χ^2^ = 163.27, *p* < 0.001), but there was no significant effect of nestling age (β = –0.0056 ± 0.0046, χ^2^ = 1.49, *p* = 0.22). Post hoc comparisons indicated significant differences among study areas. Concentrations of PFOA in nestling plasma samples from Apostle Islands National Lakeshore were greater than those from all other sites; Lake Superior southern shore nestling plasma concentrations did not differ from those at Mississippi National River and Recreation Area, but were greater than those at Pools 3 + 4, and concentrations in nestlings at upper St. Croix National Scenic Riverway and lower St. Croix National Scenic Riverway were lower than those at all other sites (all *p* < 0.05; Table 1 and Figure 2B).

#### Other analytes

We found evidence of declines for 9 of the 12 analytes reported. Declines estimated from mixed effects models ranged from 4.7 to 8.0%/yr (Table 2). Concentrations of 7 of the 12 analytes increased with increasing nestling age (Table 2).

**Table 2:** Summary of analyses for sum perfluoroalkyl substances!(∑PFAS) and 12 PFAS analytes measured in blood plasma of nestling bald eagles in Wisconsin and Minnesota

Analyte	Model type	Trend in analyte concentration	Decline^a^ (%/yr)	Significant effect of nestling age?	Increase with nestling age (%/d)^a^
∑PFAS	Mixed effects	Varied among sites		Yes (increase)	1.6
PFOS	Mixed effects	Decrease	4.8	Yes (increase)	1.9
PFDS	Frailty	Decrease		Yes (increase)	
PFDA	Frailty	None		No	
PFUnA	Mixed effects	Decrease	7.5	Yes (increase)	1.6
PFDoA	Mixed effects	Decrease	8.0	No	
PFNA	Mixed effects	None overall^b^		Yes (increase)	2.0
PFTrA	Mixed effects	Decrease	4.7	No	
PFHpS	Frailty	Decrease		Yes (increase)	
PFHxS	Frailty	Decrease		Yes (increase)	
PFTeA	Frailty	None		No	
PFOA	Frailty	Decrease		No	
PFBA	Frailty	Decrease		Yes (increase)	

^a^ Mixed effects models only. For frailty models, we report the direction of effect but percentage could not be determined.

^b^ Significant decline at LSSS of 18%/yr; however, sample size was small (*n* = 10) so result should be considered tentative.

For abbreviations, see Table 1 footnote.

## DISCUSSION

All bald eagle nestlings in our study had detectable levels of at least some PFAS analytes, with PFOS being the most abundant. Concentrations in nestlings were highest at nests on rivers influenced by urban areas, but these levels decreased over the course of our study, reflecting the national decrease in production following voluntary reductions (Lindstrom et al. 2011; Route et al. 2014a) and regulatory mandates (US Environmental Protection Agency 2002). However, elevated levels were found at relatively remote sites on Lake Superior, and the lack of a decline there warrants continued concern about the effects of these bioaccumulative, toxic compounds.

### *∑PFAS* in nestling eagle plasma samples

#### Industrialized rivers

We found that ∑PFAS concentrations in nestling plasma were highest at Mississippi National River and Recreation Area, Pools 3 + 4, and lower St. Croix National Scenic Riverway, sites that were near urban centers or immediately downstream of such urban areas on the Mississippi River and the lower St. Croix River, respectively. Associations of high concentrations of PFAS with urban areas are well known from other Great Lakes locations (in sediment: Codling et al. 2018a, 2018b; Remucal 2019; in biota: Stahl et al. 2014; Gewurtz et al. 2016). Within the Mississippi River area near our study sites, several studies have documented high levels of PFAS. Tree swallow (*Tachycineta bicolor*) nestlings at Pig's Eye Lake, a pool of the Mississippi River in St. Paul (MN, USA) and at the center of our Mississippi National River and Recreation Area study area, had ∑PFAS plasma concentrations averaging 352 and 437 ng/g in 2010 and 2011, respectively (Custer et al. 2014). Eggs of great blue herons (*Ardea herodias*) from the same location averaged 340 and 492 ng/g ∑PFAS in 2010 and 2011, respectively (Custer et al. 2013). Fish (bluegill, *Lepomis macrochirus*) collected in the Mississippi River had high concentrations of PFOS and other analytes at an urban site in Minneapolis/St. Paul, and decreasing concentrations farther downstream (Delinsky et al. 2009, 2010). Sources of PFAS within the Twin Cities urban area include the 3M Cottage Grove manufacturing plant, where PFAS were produced until 2002, the Minneapolis/St. Paul wastewater treatment plant, the St. Paul Downtown Airport, and various landfills (Route et al. 2014a).

Despite heavy contaminant loads and multiple sources of PFAS, the concentrations of ∑PFAS decreased by 10 to 11%/yr in nestling plasma collected from 2006 to 2015 at Mississippi National River and Recreation Area and lower St. Croix National Scenic Riverway, and 34%/yr at Pools 3 + 4, although this last trend should be considered tentative due to limited sampling. These trends align temporally with the discontinuation of production of PFAS at the 3M plant by 2002. Although data assessing trends of concentrations of ∑PFAS in biota from Midwestern rivers in the United States are few, those available agree with our findings. For example, ∑PFAS concentrations in great blue heron eggs from Pig's Eye Lake on the Mississippi River, which had levels among the highest ever reported for eggs in 1993, had declined by >60% by 2010/2011 (Custer et al. 2013). In 4 species of fish from Pool 2 of the Mississippi River in Minneapolis/St. Paul, MN, concentrations of PFOS (which made up approximately 84% of the total PFAS) decreased by 44–76% between 2009 and 2013 (Newsted et al. 2017).

#### Lake Superior

Eagle nestlings at Lake Superior (Apostle Islands National Lakeshore and Lake Superior southern shore) had moderately high levels of ∑PFAS, despite the relative remoteness of the location. In addition, concentrations there did not decrease during the course of our study even though the time‐series was significantly longer, with samples dating back to 1995. This is in contrast to the trend along the industrialized rivers. These patterns likely reflect the hydrological regime and contamination pathways of Lake Superior, as well as the ecology of the eagles there. Lake Superior is a deep, cold, oligotrophic lake, with a water turnover time averaging 191 yr; these characteristics allow significant bioaccumulation of persistent compounds within the sediments and biota. The human population is sparse along the shores of the Lake, but tributaries may act as point sources of PFAS because there are municipal wastewater treatment plants for each town and city along each tributary, including large population centers such as Duluth (MN, USA). Lake Superior's primary source of most persistent organic pollutants is airborne deposition (Gewurtz et al. 2008) over its 82 103‐km^2^ surface area, but for PFAS, the most important source is input from the tributaries, followed by atmospheric deposition (Scott et al. 2010; Remucal 2019).

Lake Superior bald eagles consume primarily fish during the breeding season (Kozie and Anderson 1991; Dykstra 1995; Dykstra et al. 2001), although the species they consume differ somewhat from those taken by eagles nesting on inland lakes (Dykstra 1995; Dykstra et al. 2001), and Lake Superior eagles also eat more birds, including gulls (*Larus* spp.) and double‐crested cormorants (Kozie and Anderson 1991; Dykstra 1995; Warnke et al. 2002). Fish species in the Great Lakes, in comparison with conspecifics in inland lakes, generally have higher levels of persistent organic pollutants including DDE and PCBs (Giesy et al. 1995; Sokol 2015), PBDEs (Stahl et al. 2013), and PFAS (Stahl et al. 2014). Piscivorous avian species such as gulls and cormorants, one step higher up the trophic web, accumulate those pollutants, which can be further biomagnified when eagles consume them.

The lack of a detectable long‐term trend in ∑PFAS in Lake Superior nestlings contrasts with the declining concentrations of other organic pollutants, including the legacy compounds DDE and PCBs (Dykstra et al. 2019) and PBDEs (Route et al. 2014b). The declining concentration of PBDEs is particularly informative, because PBDE use was terminated much more recently (2004; US Environmental Protection Agency 2006) than that of DDE and PCBs (in the 1970s). The contrast between PFAS and the legacy contaminants may reflect differences in primary sources (atmospheric deposition for most persistent organic pollutants but via tributaries for PFAS; Gewurtz et al. 2008; Scott et al. 2010; Remucal 2019). The lack of a long‐term trend for ∑PFAS also contrasts with other reports for the Great Lakes. For example, ∑PFAS concentrations in Great Lakes herring gull (*Larus argentatus*) eggs decreased from 1990 to 2010 (Gebbink et al. 2011). In general, concentrations of ∑PFAS in sediment and biota of Lake Superior and the other upper Great Lakes increased until approximately 2000, and has then decreased since that time (Remucal 2019). We suggest that the absence of a decline in ∑PFAS concentrations at Lake Superior may reflect the importance of long‐chain perfluoroalkyl carboxylates (PFCAs) there (see discussion in section *Individual PFAS analytes in nestling eagle plasma samples*), although a lack of federal regulation of most PFAS also likely contributes.

#### Remote river site (upper St. Croix National Scenic Riverway)

The upper St. Croix River is remote, narrow, and relatively uninfluenced by anthropogenic impacts (Dykstra et al. 2019). In the present study, the ∑PFAS concentrations in nestling plasma from upper St. Croix National Scenic Riverway did not show a temporal trend, although concentrations were low throughout the study. In our previous study, we found moderate evidence of a decline in ∑PFAS concentrations at upper St. Croix National Scenic Riverway from 2006 to 2011 (80% probability of decline; Route et al. 2014a). The differences likely reflect the longer time series of the present study or the different analyses.

### Individual PFAS analytes in nestling eagle plasma samples

Overall, PFOS and PFDS made up the majority of the total PFAS in nestling plasma samples in our study, confirming our earlier results based on a shorter time‐series (Route et al. 2014a). Concentrations of these analytes paralleled ∑PFAS in being greatest in nestlings from the industrialized rivers, Mississippi National River and Recreation Area, Pools 3 + 4, and lower St. Croix National Scenic Riverway, and lowest in nestlings from upper St. Croix National Scenic Riverway.

Even so, the nestlings at the most remote site, Apostle Islands National Lakeshore, had the highest concentrations for 7 of the 12 analytes, including some of the longer chain PFCAs (such as PFNA, PFUnA, and PFDA; Table 1). Some of these long‐chain PFCAs are associated with decreased reproduction in wild birds (black‐legged kittiwakes [*Rissa tridactyla*]; Tartu et al. 2014), and PFCA concentrations are generally increasing in wild birds (Holmström et al. 2010; Ahrens et al. 2011; Gebbink et al. 2011; Miller et al. 2015; Vorkamp et al. 2019). Long‐chain PFCAs tend to be relatively more abundant than perfluoroalkyl sulfonates (PFSAs) in western Great Lakes fish and birds (Letcher et al. 2015; Remucal 2019), which agrees with our analysis, in which the most abundant PFAS in nestlings at Lake Superior sites (after PFOS) were all the longer chained PFCAs (Table 1). Concentrations of PFCAs have generally remained constant or increased in Great Lakes herring gull eggs (Letcher et al. 2015; Remucal 2019). The importance of long‐chain PFCAs at Lake Superior compared with the Mississippi River and lower St. Croix River likely reflects different sources of PFAS, such as more long‐range atmospheric deposition (Route et al. 2014a; Costantini et al. 2019), as well as slower removal from the ecosystem at this large lake.

Over all the study areas combined, we found decreasing concentrations in nestling plasma from 2006 to 2015 for 9 of the 12 analytes (Table 2). Such declines mirror the decreasing concentrations of ∑PFAS in our samples from the industrialized rivers.

#### PFOS

Concentrations of PFOS made up the majority of ∑PFAS in nestling bald eagles at all our study areas, although PFOS was much more important at sites along the industrialized rivers than at Lake Superior, where long‐chain PFCA analytes such as PFNA contributed significantly to the nestlings' total PFAS burden (Table 1; Elliott et al. 2019). Likewise, PFOS made up the majority of ∑PFAS in avian samples in many locations in the Great Lakes region (Letcher et al. 2015; Remucal 2019; Wu et al. 2020), with the percentage of the total burden similarly depending on waterbody type and distance from point sources. Tree swallow eggs and plasma from sites near point sources on the Mississippi River had the greatest percentage contribution by PFOS (>95% in eggs), whereas those near other known point sources were somewhat lower (67–87% in plasma), and those from isolated lakes without point sources were much lower (30–40%; Custer et al. 2019). In general, PFOS concentrations within the Great Lakes basin are higher near urban/industrial areas (Remucal 2019); the predominance of PFOS in avian samples may be an indicator of the proximity to point sources (Custer et al. 2019).

Overall concentrations of PFOS in nestling plasma declined from 2006 to 2015 in our study. Likewise, concentrations of PFOS also decreased dramatically in great blue heron eggs from the Mississippi River between 1993 and 2010/2011 (Custer et al. 2013), in Great Lakes herring gull eggs between 1990 and 2010 (Remucal 2019), and in tawny owl (*Strix aluco*) eggs (Ahrens et al. 2011). Concentrations of PFOS failed to decrease in eggs of peregrine falcons from remote Greenland (1986–2014; Vorkamp et al. 2019) and Sweden (Holmström et al. 2010), eggs of ospreys (*Pandion haliaetus*) in Sweden (Eriksson et al. 2016), and eggs of white‐tailed eagles (*Haliaeetus albicilla*) from Sweden (Faxneld et al. 2016). Differing trends are apparently related to differing locations (Eriksson et al. 2016), but few recent studies show increasing concentrations of PFOS (Vorkamp et al. 2019; but see Groffen et al. 2019 for an exception), which likely reflects the discontinuation of PFOS production in the first few years of this century (Remucal 2019).

Concentrations of PFOS below approximately 1000 μg/L in blood serum and eggs are thought to have no effect in avian species, based on laboratory toxicity testing in ducks and quail, while lowest observable effect thresholds were approximately 1700 μg/L for plasma and eggs (Newsted et al. 2005). Although no mean nestling PFOS concentrations in our study approached these thresholds, individual nestlings at Mississippi National River and Recreation Area, Pools 3 + 4, and lower St. Croix National Scenic Riverway exceeded these concentrations, suggesting that some impairment may have occurred. Similarly high concentrations occurred in individual Midwestern bald eagle nestlings in the early 1990s (Kannan et al. 2001). Primarily in laboratory studies, PFOS has been associated with negative effects on liver, lungs, and kidney, changes in body mass, reduction in normal activity, and developmental toxicity (Stahl et al. 2011). In the field, analyses have had mixed results. Mean concentrations of total PFAS (primarily PFOS) as low as 150 ng/g in tree swallow eggs were apparently associated with lower hatching success (Custer et al. 2012, 2014); however, in later work, tree swallows with much higher loads of PFAS reproduced well, prompting the researchers to conclude that the earlier study may have been confounded by additive effects of other contaminants (Custer et al. 2019). Similarly, in Belgium, very high concentrations of PFAS had somewhat limited effects on reproduction in great tits (Groffen et al. 2019). However, in black‐legged kittiwakes, reduced hatching success was associated with higher concentrations of PFDoA, one of the long‐chain PFCAs (Tartu et al. 2014).

#### PFDS

Together with PFOS, PFDS made up most (>90%) of the ∑PFAS in plasma of bald eagle nestlings in our earlier report (Route et al. 2014a), and was similarly important in the present study for nestlings on the industrialized rivers (Mississippi National River and Recreation Area, Pools 3 + 4, and lower St. Croix National Scenic Riverway), although not for those at Lake Superior (Apostle Islands National Lakeshore and Lake Superior southern shore) or the remote upper St. Croix National Scenic Riverway (Table 1). Perfluorodecanesulfonate was detected in 100% of Caspian tern eggs, herring gull eggs, and bald eagle eggs from Great Lakes sites, and was the second most abundant of the perfluorinated sulfonates, following PFOS (gulls: Letcher et al. 2015; terns: Su et al. 2017; eagles: Wu et al. 2020). As a long‐chain compound, PFDS is more likely to be bioaccumulative and toxic (Conder et al. 2008), but has been less studied than PFOS and PFOA, despite its prevalence and apparent association with urban areas (Route et al. 2014a; Letcher et al. 2015; Gewurtz et al. 2016).

In our study, overall PFDS concentrations in nestling eagle plasma decreased over time. Concentrations of PFDS in Great Lakes herring gull eggs at 7 colonies also declined over the period from 1990 to 2010 (Gebbink et al. 2011). Similarly, concentrations of PFDS also decreased by 76% in great blue heron eggs from the Mississippi River between 1993 and 2010/2011 (Custer et al. 2013). Outside of the Great Lakes region, the temporal trends are generally similar. In peregrine falcon eggs from Greenland, PFDS made up only a small percentage of the overall PFAS load (which was dominated by PFOS), and decreased at a rate of 0.6% annually from 1986 to 2014 (Vorkamp et al. 2019). In tawny owl eggs collected in Norway, PFDS concentrations decreased 6.6% annually from 1997 to 2009, after increasing from 1986 to 1997 (Ahrens et al. 2011). Concentrations of PFDS in peregrine falcon eggs from Sweden increased from 1974 to approximately 2002, but then showed no trend until 2007 (Holmström et al. 2010). In contrast to these studies showing declines, a study of great tit eggs at a highly contaminated site in Belgium found increases in PFDS and PFOS concentrations between 2011 and 2016 (Groffen et al. 2019).

#### PFOA

Even though PFOA is more abundant than PFOS in the surface water of the Great Lakes (Scott et al. 2010; Remucal 2019), bald eagle nestlings in all study areas had fairly low concentrations of this compound in plasma. Relative to PFOS, PFOA has a low bioaccumulative potential (Kannan et al. 2005; Remucal 2019; Bursian et al. 2020), and is considered less toxic (Stahl et al. 2011; Bursian et al. 2020). Our results paralleled those of some other biota within the Great Lakes basin. Concentrations of PFOA were detected in only 12% of Great Lakes fish composite samples in which PFOS was detected in 100% (Stahl et al. 2014). However, in plasma samples of tree swallow nestlings, PFOA was detected in 100% of samples from a highly contaminated site in Oscoda, Michigan (USA); PFOA was the second most abundant PFAS at sites on the Upper Mississippi River, and was primarily responsible for the variation in PFAS analyte profiles among sites and between years (Custer et al. 2014, 2019).

Overall PFOA concentrations in our study decreased over time, confirming our earlier results (Route et al. 2014a). Similarly, PFOA concentrations have also declined in Great Lakes herring gull eggs (Gebbink et al. 2011; Remucal 2019).

### Nestling age

Nestling age significantly influenced nestling plasma concentrations of 7 of the 12 analytes we studied. For all these analytes, concentrations increased as nestlings aged, generally by 1 to 2%/d (Table 2). These findings expand on our previous results that used only the earlier samples, in which we found relationships with nestling age for 3 of 12 analytes (2 increasing trends, 1 decreasing; Route et al. 2014a); the differences likely relate to the greater number of samples and longer time‐series of the present study. The bioaccumulative properties of the analytes, combined with the increasing exposure time and increasing dietary input as the nestlings age, likely explain these trends. It is unlikely that these relationships reflect a broader correlation to date (Julian day), such as might occur if eagles routinely switched from one prey type to another (more contaminated) type as the season progressed, because the wide geographic and hydrologic range of our study areas resulted in hatch dates that varied by approximately 1 mo on average from south to north (W. Route, unpublished data), and prey species that differed among waterbody types (W. Route, unpublished data).

Studies assessing the relationship of contaminant concentrations in raptor plasma to nestling age are limited. However, PFOS concentrations in nestling white‐tailed eagle and northern goshawks (*Accipiter gentilis*) sampled repeatedly also increased as the nestlings aged (Bustnes et al. 2013). Researchers concluded that the trends reflected dietary input of this PFAS, and that the magnitude of the increase indicated relatively high intakes of PFOS via diet (Bustnes et al. 2013). In a study similar to ours in which nestling age was considered as a covariate in analysis of PFAS concentrations, total PFAS concentrations in plasma samples from white‐tailed eagle nestlings increased significantly with age (range, 44–87 d), and researchers also concluded that plasma trends represented continuing dietary exposure to PFAS (Løseth et al. 2019). These results and ours indicate that researchers and managers should consider nestling age when using nestling plasma to assess concentrations of PFAS. We recommend further studies on this topic, including repeated quantifications of PFAS loads as individual animals age.

## CONCLUSIONS

Nestling blood plasma concentrations of ∑PFAS differed among study areas and were highest at the 3 industrialized river sites (Pools 3 + 4, Mississippi National River and Recreation Area, and lower St. Croix National Scenic Riverway), moderately high at Lake Superior sites (Apostle Islands National Lakeshore and Lake Superior southern shore), and lowest at upper St. Croix National Scenic Riverway. Temporal trends in ∑PFAS also differed among study areas; concentrations decreased at Pools 3 + 4, Mississippi National River and Recreation Area, and lower St. Croix National Scenic Riverway, but not at the most remote sites, the upper St. Croix River and Lake Superior. Overall, PFOS was the most abundant analyte at all study areas, and PFDS the second most abundant at industrialized river sites though not at Lake Superior, where other analytes were more important. One Lake Superior site (Apostle Islands National Lakeshore) had the highest concentrations for 7 of the 12 analytes, including some of the longer chain PFCAs such as PFNA, PFUnA, and PFDA. Over the entire study area, concentrations in nestling plasma decreased from 2006 to 2015 for 9 of the 12 analytes, but nestling age significantly influenced plasma concentrations of ∑PFAS and 7 of the 12 analytes, indicating that age should be included in future assessments.

## Supplemental Data

The Supplemental Data are available on the Wiley Online Library at https://doi.org/10.1002/etc.4864. The Data include 4 figures showing: (S1) map of study area, (S2) relationship between ∑PFAS in nestling plasma and nestling age, (S3) mean ∑PFAS concentrations in plasma of nestling bald eagles at 6 study areas, and (S4) mean PFOS concentrations in plasma of nestling bald eagles at 6 study areas. The 3 tables show: (S1) number of nestling plasma samples measured for PFAS by year at 6 study areas, (S2) names and abbreviations of PFAS analytes measured, and (S3) number of nestling plasma samples by analyte and the percentage of samples that were below the laboratory's limits of quantification.

## Author Contributions Statement

The manuscript was written through contributions of all authors. All authors have approved the final version of the manuscript.

## Supporting information

This article includes online‐only Supplemental Data.

Supporting information.

## Data Availability

The data included in this manuscript are publicly available free of charge from the National Park Service Data Store for direct download at: https://irma.nps.gov/DataStore/DownloadFile/606817. Data, associated metadata, and calculation tools are available from the corresponding author (billroute2@gmail.com).
