# Supplementary material for: Trends and Patterns of Perfluoroalkyl Substances in Blood Plasma Samples of Bald Eagle Nestlings in Wisconsin and Minnesota, USA
Source: Environ Toxicol Chem. 2020 Oct 20;40(3):754–66. doi: 10.1002/etc.4864 (PMC7984356; doi:10.1002/etc.4864)
Supplement: Supplementary file 1 — Supporting information. [file ETC-40-754-s001.docx]

Supporting Information

Trends and Patterns of Perfluoroalkyl Substances in BLOOD PLASMA SAMPLES of Bald Eagle Nestlings in Wisconsin and Minnesota, USA

Cheryl R. Dykstra^a^, William T. Route^b^, and Kelly A. Williams^c^

1. Raptor Environmental, 7280 Susan Springs Drive, West Chester, Ohio, 45069, USA
2. U.S. National Park Service, Great Lakes Inventory and Monitoring Network, Ashland, Wisconsin, 54806, United States. Current address: Northwoods Wildlife Consulting, 29580 County Highway C, Washburn, Wisconsin, 54891, USA
3. Department of Biological Sciences, 430 Irvine Hall, Ohio University, Athens, Ohio, 45701, United States.

Figure S1. Map of the 6 study areas in Wisconsin and Minnesota where bald eagle nestlings were sampled for PFAS concentrations.


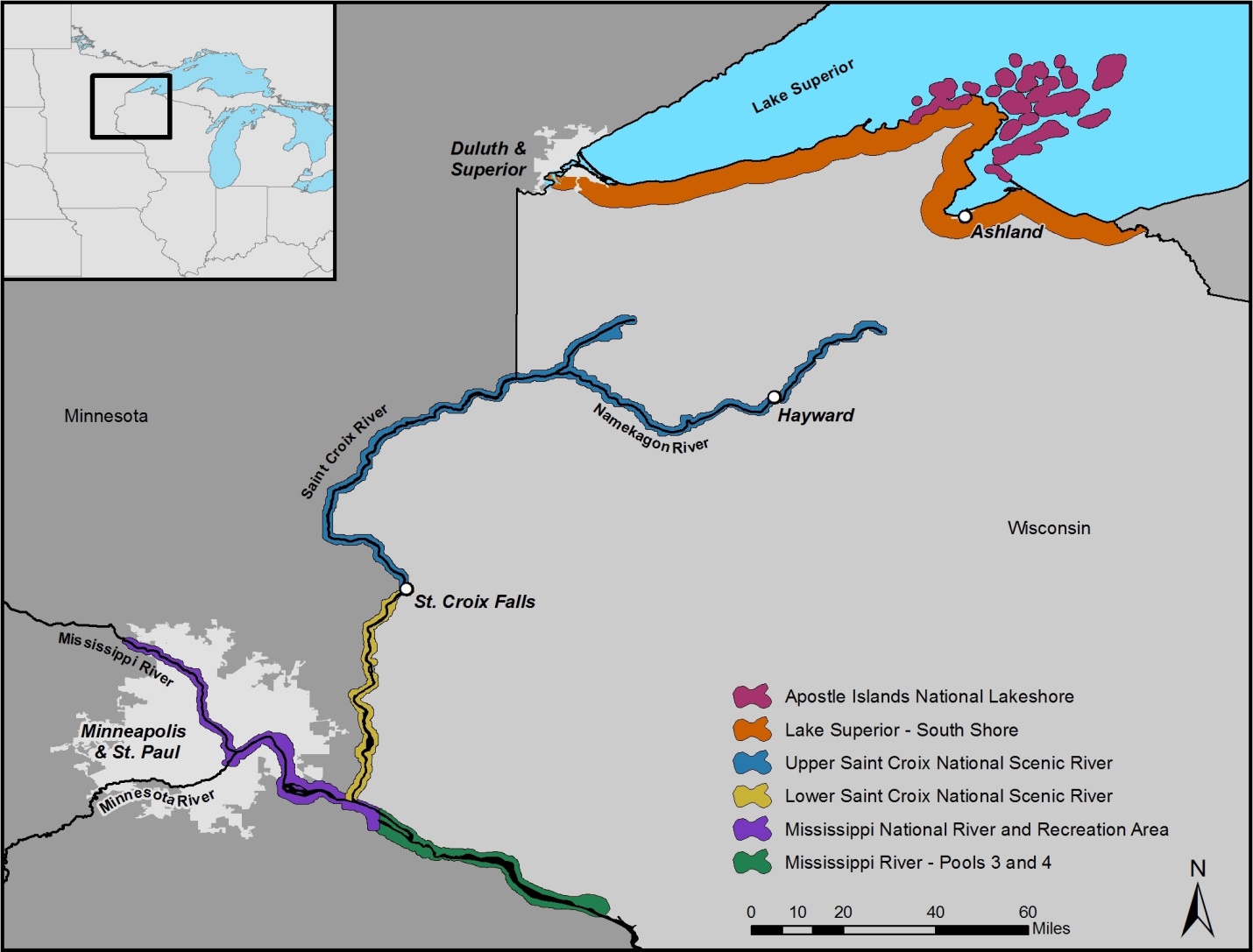


Figure S2. Example of the relationship between nestling age and ΣPFAS in plasma of nestling bald eagles sampled at one study area on Lake Superior. APIS = Apostle Islands.


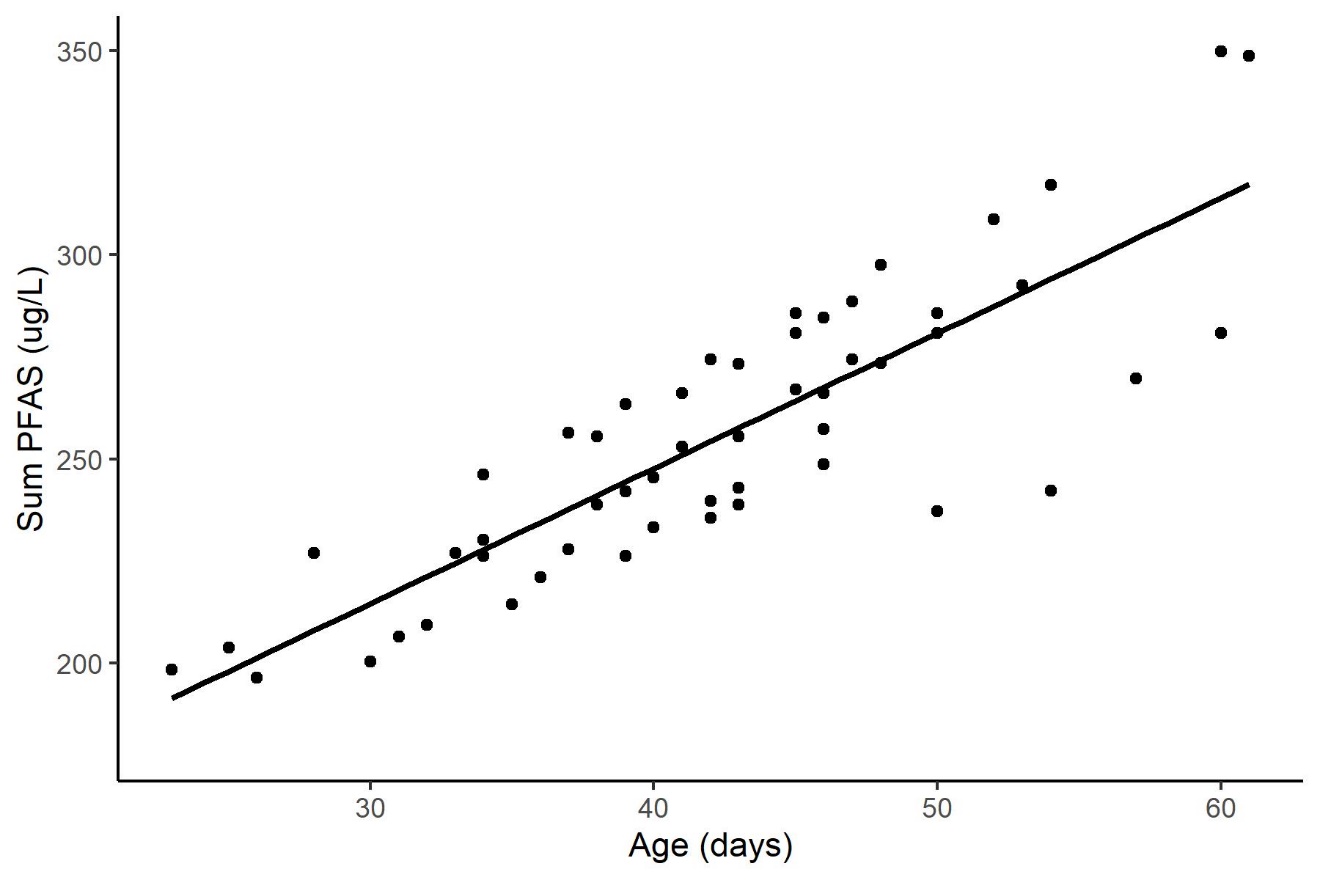


Figure S3. PFAS concentrations (log_10_ ΣPFAS) in plasma of nestling bald eagles sampled at 6 study areas in northern Wisconsin and adjacent areas of Minnesota, 2006 to 2015. APIS = Apostle Islands, LSSS = the south shore of Lake Superior in Wisconsin, USACN=upper Saint Croix National Scenic River, LSACN = lower Saint Croix National Scenic River, MISS = Mississippi National River and Recreation Area, and Pools3+4 = pools 3 and 4 of the Mississippi River. Least-square means calculated from the model with study area × year interaction, nestling age as a covariate, and territory as a random factor.


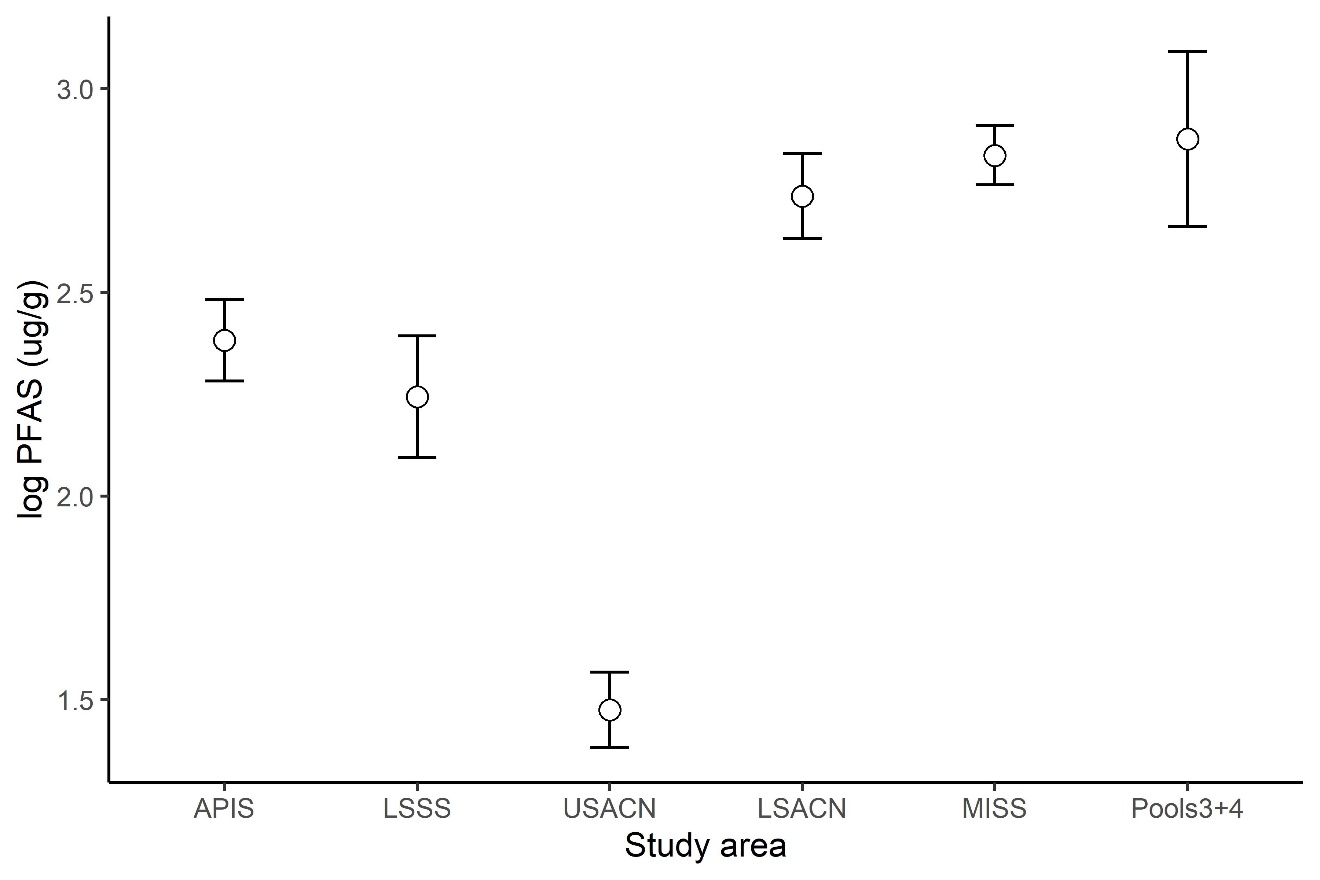


Figure S4. PFOS concentrations (log_10_ PFOS) in plasma of nestling bald eagles sampled at 6 study areas in northern Wisconsin and adjacent areas of Minnesota, 2006 to 2015. APIS = Apostle Islands, LSSS = the south shore of Lake Superior in Wisconsin, USACN=upper Saint Croix National Scenic River, LSACN = lower Saint Croix National Scenic River, MISS = Mississippi National River and Recreation Area, and Pools3+4 = pools 3 and 4 of the Mississippi River. Least-square means estimated from the mixed effects model with study area × year interaction, nestling age as a covariate, and territory as a random factor.


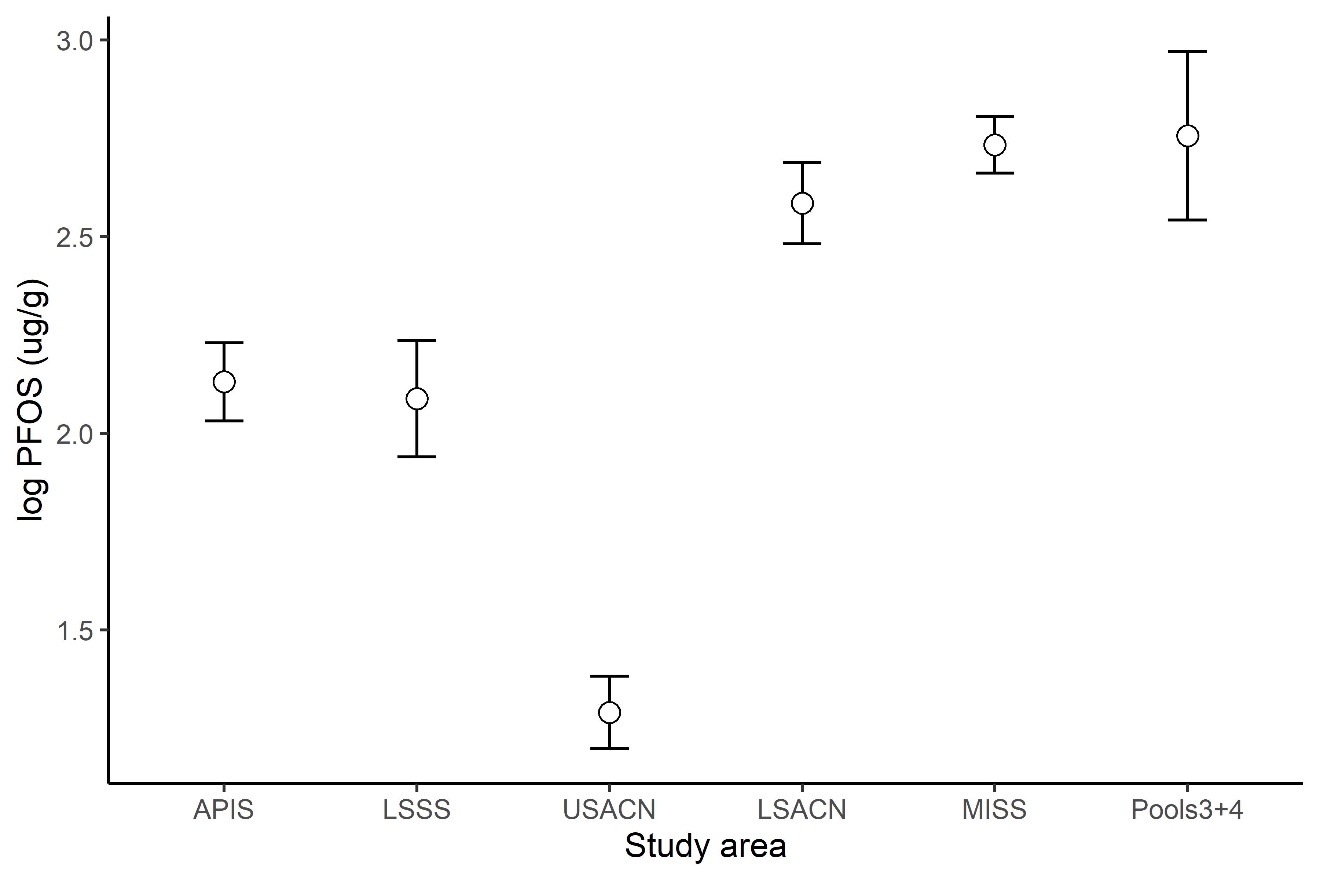


Table S1. Number of nestling plasma samples measured for PFAS by year at 6 study areas in Wisconsin and Minnesota

|  | Study area | | | | | |  |
| --- | --- | --- | --- | --- | --- | --- | --- |
| Year | APIS | LSACN | LSSS | MISS | Pools | SACN | Total by year |
| 1995 | 0 | 0 | 3 | 0 | 0 | 0 | 3 |
| 1996 | 0 | 0 | 5 | 0 | 0 | 0 | 5 |
| 1997 | 0 | 0 | 1 | 0 | 0 | 0 | 1 |
| 1998 | 1 | 0 | 1 | 0 | 0 | 0 | 2 |
| 2000 | 1 | 0 | 0 | 0 | 0 | 0 | 1 |
| 2002 | 2 | 0 | 0 | 0 | 0 | 0 | 2 |
| 2006 | 8 | 3 | 0 | 10 | 0 | 11 | 32 |
| 2007 | 6 | 4 | 6 | 11 | 0 | 8 | 35 |
| 2008 | 5 | 7 | 4 | 15 | 15 | 0 | 46 |
| 2009 | 0 | 9 | 0 | 18 | 12 | 0 | 39 |
| 2010 | 9 | 13 | 0 | 23 | 4 | 11 | 60 |
| 2011 | 9 | 12 | 1 | 20 | 2 | 12 | 56 |
| 2014 | 10 | 7 | 0 | 21 | 0 | 10 | 48 |
| 2015 | 10 | 9 | 5 | 23 | 0 | 12 | 59 |
| **TOTAL** | **61** | **64** | **26** | **141** | **33** | **64** | **389** |

Table S2. Names and abbreviations of PFAS analytes measured in bald eagle nestlings in Wisconsin and Minnesota

| PFAS type | Abbreviation | Analyte name | Long-chain or short-chain^a^ (carbon chain length) |
| --- | --- | --- | --- |
| Perfluoroalkyl sulfonates (PFSA) | PFHxS | Perfluorohexanesulfonate | Long (6) |
|  | PFHpS | Perfluoroheptanesulfonate | Long (7) |
|  | PFOS | Perfluorooctanesulfonate | Long (8) |
|  | PFDS | Perfluorodecanesulfonate | Long (10) |
| Perfluoroalkyl carboxylates (PFCA) | PFBA | Perfluorobutanoate | Short (4) |
|  | PFOA | Perfluorooctanoate | Moderate (8) |
|  | PFNA | Perfluorononanoate | Long (9) |
|  | PFDA | Perfluorodecanoate | Long (10) |
|  | PFUnA | Perfluoroundecanoate | Long (11) |
|  | PFDoA | Perfluorododecanoate | Long (12) |
|  | PFTrA | Perfluorotridecanoate | Long (13) |
|  | PFTeA | Perfluorotetradecanoate | Long (14) |

1. PFSAs are designated as long if ≥6 carbons, and PFCAs as long if ≥8 carbons (as in Remucal 2019); however, Remucal (2019) also describes PFOA as a “moderate-chain” PFCA, so we adopt that terminology above.

Table S3. Number of nestling plasma samples measured for PFAS analytes and the percent of samples that were below the laboratory’s limits of quantification; PFPA, PFHxA, and PFHpA were not analyzed individually due to high percentage of samples <LOQ

| Analyte^a^ | Number of samples^b^ | Percent of samples <LOQ |
| --- | --- | --- |
| ΣPFAS | 389 | 0 |
| PFOS | 389 | 0 |
| PFDS | 322 | 1.2 |
| PFDA | 322 | 0.9 |
| PFUnA | 322 | 0 |
| PFDoA | 322 | 0.3 |
| PFNA | 322 | 0 |
| PFTrA | 322 | 0 |
| PFHpS | 322 | 6.2 |
| PFHxS | 322 | 6.2 |
| PFTeA | 322 | 1.9 |
| PFOA | 389 | 18.5 |
| PFBA | 322 | 46 |
| PFPA | 322 | 98.4 |
| PFHxA | 322 | 90.4 |
| PFHpA | 322 | 97.8 |

1. Analyte names and abbreviations shown in Table S2.
2. Most analytes were missing 67 values because only PFOS and PFOA were measured in 2006 and 2007 (*n* = 67 nestlings).
